# Traditional and Modern Predictors of Atherosclerotic Cardiovascular Disease in Patients with T2D and MASLD

**DOI:** 10.3390/diagnostics16111607

**Published:** 2026-05-25

**Authors:** Cosmina-Theodora Vulpescu (Diaconu), Marius-Costin Chitu, Teodor Salmen, Anca Pantea Stoian, Cristian Guja

**Affiliations:** 1 Doctoral School, Carol Davila University of Medicine and Pharmacy, 020021 Bucharest, Romania; cosmina-theodora.diaconu@drd.umfcd.ro (C.-T.V.);; 2 Pitesti County Emergency Hospital, 110084 Arges, Romania; 3 Department of Diabetes, Nutrition and Metabolic Diseases, “Carol Davila” University of Medicine and Pharmacy, 020021 Bucharest, Romania; anca.stoian@umfcd.ro (A.P.S.); cristian.guja@hotmail.com (C.G.)

**Keywords:** metabolic dysfunction-associated steatotic liver disease (MASLD), non-alcoholic fatty liver disease (NAFLD), atherosclerotic cardiovascular disease (ASCVD), type 2 diabetes (T2D), coronary artery calcification (CAC), HbA1c, Fibrosis-4 Index (FIB-4), cardiometabolic risk, GLP-1 receptor agonists, SGLT2 inhibitors, non-invasive fibrosis tests (NIT)

## Abstract

**Background/Objectives:** Metabolic dysfunction-associated steatotic liver disease (MASLD) is highly prevalent in patients with type 2 diabetes (T2D) and is associated with increased cardiovascular risk. However, the relative contribution of traditional cardiometabolic risk factors (CMRFs), hepatic fibrosis markers, and antidiabetic therapies to atherosclerosis remains unclear. **Methods:** We conducted a cross-sectional study including 46 patients with T2D and MASLD. Atherosclerotic cardiovascular disease (ASCVD) was defined as the presence of carotid atheromatosis, stroke, peripheral arterial disease, or ischemic heart disease, as assessed by imaging-based parameters. Clinical, metabolic, and treatment-related variables were analyzed, including age, Hemoglobin A1c (HbA1c), lipid profile, hepatic fibrosis indices such as Fibrosis-4 index (FIB-4), and antidiabetic therapies (sodium–glucose cotransporter-2 inhibitors (SGLT2is), glucagon-like peptide-1 receptor agonists (GLP-1 RAs), and insulin). Multivariable regression models and receiver operating characteristic (ROC) curve analyses were used to evaluate associations and discriminative performance. **Results:** Traditional CMRFs were more strongly associated with ASCVD than hepatic fibrosis markers or antidiabetic therapies. Age was associated with ASCVD in several exploratory models, although this association was attenuated in the fully adjusted model. HbA1c showed the highest discriminative performance (AUC 0.77), indicating that chronic glycemic exposure is a major determinant of vascular disease in this cohort. In contrast, FIB-4 was not associated with ASCVD and did not improve model performance. Antidiabetic therapies, including SGLT2i and GLP-1 RAs, were not independently associated with ASCVD. Insulin therapy was more frequent among patients with ASCVD, but was not independently associated after adjustment. **Conclusions:** In patients with T2D and MASLD, ASCVD appears to be associated with traditional CMRFs, particularly chronic glycemic exposure, rather than hepatic fibrosis markers or treatment status. These findings highlight the central role of metabolic control in cardiovascular risk and suggest that the contribution of liver-related markers and therapeutic interventions may be more relevant in longitudinal settings.

## 1. Introduction

Type 2 diabetes (T2D) represents a major cardiometabolic risk factor (CMRF) and plays a central role in the development and progression of metabolic dysfunction-associated steatotic liver disease (MASLD) [1,2]. MASLD is the most common chronic liver disease worldwide, with an estimated global prevalence of 25–30%, reaching up to 38% in the most recent surveys [3]. It is characterized by the presence of hepatic steatosis in the absence of alcohol consumption or viral hepatitis. MASLD replaced the previously used term non-alcoholic fatty liver disease (NAFLD), following the recent consensus for increased accuracy and improved definition [4].

Beyond liver-related complications, MASLD is increasingly recognized as a multisystem disease. While progressive forms such as metabolic dysfunction-associated steatohepatitis (MASH) are associated with advanced fibrosis and cirrhosis, cardiovascular disease (CVD) remains the leading cause of mortality in this population [5,6]. Patients with MASLD, particularly those with T2D, exhibit a substantially increased risk of atherosclerotic cardiovascular disease (ASCVD) [7], driven by shared mechanisms including insulin resistance, chronic low-grade inflammation, endothelial dysfunction, and pro-atherogenic dyslipidemia [8,9,10]. Hepatic and visceral ectopic fat promote inflammatory cytokine release, impair nitric oxide availability, and alter lipid metabolism, leading to increased triglycerides, small, dense Low-Density Lipoprotein cholesterol (LDL-cholesterol) particles, and reduced High-Density Lipoprotein cholesterol (HDL-cholesterol), all of which favor plaque formation and vascular injury [10]. However, because MASLD overlaps substantially with metabolic syndrome and T2D, the extent to which ASCVD risk is independently related to liver disease itself, rather than to shared CMRFs, remains uncertain. This controversy is further amplified by differences in NAFLD/MASLD definitions, study populations, fibrosis assessment methods, cardiovascular endpoints, and the use of cross-sectional versus longitudinal study designs.

A recent study using the new MASLD nomenclature reported that, after adjustment for CMRFs, MASLD was no longer independently associated with cardiovascular mortality [11]. In contrast, earlier studies based on the NAFLD definition identified hepatic steatosis as an independent predictor of cardiovascular outcomes [12]. These findings highlight the need to more clearly delineate the relative contributions of traditional risk factors and liver-related markers in cardiovascular risk stratification.

The recent global consensus recommendations emphasize a fibrosis-centered approach to MASLD management, with the Fibrosis-4 index (FIB-4) as a first-line screening tool for risk stratification, owing to its accessibility, cost-effectiveness, and ability to reliably exclude advanced liver disease [13].

Attention has increasingly shifted from hepatic steatosis to fibrosis severity as a potential determinant of cardiovascular risk in MASLD. In this context, FIB-4 is used as a potential tool for cardiovascular risk stratification. Several longitudinal studies have reported an association between higher FIB-4 values and progression of Coronary artery calcification (CAC) [14], a marker of subclinical atherosclerosis or incident cardiovascular events, including heart failure, even after adjustment for traditional risk factors [15]. However, these associations are generally modest, and the additional value of hepatic fibrosis markers beyond traditional cardiometabolic risk factors remains controversial, particularly in patients with established T2D.

In parallel, newer antidiabetic therapies, including sodium–glucose cotransporter-2 inhibitors (SGLT2i) and glucagon-like peptide-1 receptor agonists (GLP-1 RA), have shown cardiovascular and metabolic benefits, raising the question of whether treatment-related factors may also influence atherosclerotic burden in patients with MASLD and T2D.

Despite growing interest in the link between MASLD and cardiovascular risk, data comparing the relative associations of traditional CMRFs, hepatic fibrosis markers, and antidiabetic therapies to ASCVD in patients with T2D and MASLD remain limited, particularly in real-life clinical settings.

Therefore, the aim of this study was to evaluate the association between traditional cardiometabolic risk factors, hepatic non-invasive tests (NIT), and antidiabetic therapies with ASCVD in patients with T2D and MASLD, and to assess their relative predictive value.

## 2. Materials and Methods

This cross-sectional observational study was carried out according to the STROBE (Strengthening the Reporting of Observational Studies in Epidemiology) guidelines, which were the framework on which this section was developed [16].

### 2.1. Study Population

Consecutive patients presented at the same diabetologist from the Outpatient Department of NC Paulescu National Institute of Diabetes Mellitus, Nutrition and Metabolic Disorders, Bucharest, Romania, between 13 July 2023 and 16 March 2026, with 5 outpatient days a week, were evaluated per day for inclusion in the study.

### 2.2. Inclusion and Exclusion Criteria

#### 2.2.1. Inclusion Criteria

All participants were adults with a previously established diagnosis of T2D for at least 6 months, a complete biologic profile, and a diagnosis of MASLD. The diagnosis of MASLD was established based on abdominal ultrasonography (US), in accordance with the multisociety Delphi consensus (1), the presence of T2D fulfilled the requirement of at least one cardiometabolic risk factor in all patients.

#### 2.2.2. Exclusion Criteria

Patients were excluded if they did not meet the MASLD diagnostic criteria according to the multisociety Delphi consensus (1) or if they had secondary causes of hepatic steatosis.

These included alcohol-associated liver disease (ALD), defined as an average daily alcohol consumption >50 g for females or >60 g for males; metabolic dysfunction-associated steatotic liver disease with increased alcohol intake (MetALD), defined as alcohol consumption >20 g/day for females or >30 g/day for males; specific aetiologies of hepatic steatosis (including drug-induced liver injury, monogenic diseases, and chronic viral hepatitis B or C infection); and cryptogenic hepatic steatosis.

### 2.3. Clinical Assessment

A complete medical history was conducted, focusing on metabolic risk factors, alcohol intake, current and prior medication use, and the identification of potential secondary causes of hepatic steatosis. A history of T2D duration, heredity and complications (neuropathy, retinopathy, chronic kidney disease, atherosclerotic cardiovascular disease (ASCVD)—ischemic heart disease, stroke, arterial peripheric disease, and the presence of carotid atheromatosis) and comorbidities (HBP (High Blood Pressure), dyslipidemia, hepatic steatosis, Metabolic Syndrome, left ventricular hypertrophy) was attained.

Demographic characteristics (age, gender, environment) were collected. Clinical characteristics (weight, height, and waist circumference) were assessed while patients were barefoot and wearing light clothing. Body mass index (BMI) was calculated as weight/height^2^ (kg/m^2^), and waist circumference (WC) was measured at the midpoint between the inferior rib margin and superior iliac crests.

Regarding medication, of interest were antidiabetic medications (GLP-1 RA, SGLT-2i, Dipeptidyl Peptidase-4 Inhibitors (DPP4i), sulfonylurea, and insulin treatment). Also, the patients were asked whether they had taken HBP-lowering medication (beta-blockers, calcium-channel blockers, ACEIs/ARBs) for CV apparatus (statins, ezetimibe, fibrates, iPCSK9) in the last 3 months.

Blood Pressure (BP) was measured after 10 min of rest, with the patient sitting in a back-supporting position, with their legs uncrossed, feet flat on the floor, and arm at heart level. 2 measurements were obtained, and the mean was used in the current study.

Some patients were evaluated through ankle–brachial index measurement. The patients sat in a supine position, and using a portable Doppler ultrasound and blood pressure cuffs, systolic BP was measured in both arms and legs. The ankle–brachial index was calculated as the ratio of ankle to brachial systolic BP.

### 2.4. Biological Assessment

The biological assessment included HbA1c (normal values <5.7%), glycaemia (normal range: 70–100 mg/dL), total cholesterol (normal range: 50–200 mg/dL), HDL-cholesterol (normal value, <55 mg/dL), LDL-cholesterol (normal value according to cardiovascular risk score, mostly <55 mg/dL), triglycerides (normal range: 50–150 mg/dL), uric acid (normal value 2.40–5.7 mg/dL), creatinine (normal value 0.50–1.10 mg/dL), TGO (normal value <32 UI/L), TGP (normal value <33 UI/L), GGT (normal value 5.00–36.00 UI/L), eGFR (normal value >60 mL/min/17.3 m^2^), urea (normal value 7.40–49.00 mg/dL), haemoglobin (normal value 11.70–15.00 g/dL), hematocrit (normal value 36.00–48.00 mg/dL), platelets (normal value 150.00–450.00/uL), urinary albumin–creatinine ratio (UAC normal value <30 mg/g creatinine).

### 2.5. MASLD Assessment

The diagnosis of MASLD was established based on abdominal ultrasonography (US), performed by the same operator using a Philips HD11XE system (Philips, Washington, DC, USA) equipped with a C5-2 convex array probe (2.0–5.0 MHz). Hepatic steatosis was diagnosed based on increased hepatic echogenicity relative to the renal cortex (hepatorenal contrast), posterior beam attenuation, and blurring of intrahepatic vascular structures, in accordance with standard US criteria. After the confirmation of hepatic steatosis, the diagnosis of MASLD was made based on the presence of T2D, as per the cardiometabolic criteria. Other causes of secondary steatosis, such as ALD, MetALD, drug-induced liver injury, monogenic diseases, and chronic viral hepatitis B or C infection, and cryptogenic hepatic steatosis, were excluded.

### 2.6. Ethical Approval

This study was conducted in accordance with the Helsinki Declaration and received ethical approval from the Ethical Committee of the NC Paulescu National Institute of Diabetes Mellitus, Nutrition and Metabolic Disorders, Bucharest, Romania, number 3196, dated 7 July 2023.

### 2.7. Statistical Analysis

Statistical analysis was performed using PSPP 2.1.1. and Excel 2021 software. Continuous variables were assessed for normality using the Shapiro–Wilk test and are presented as mean, standard deviation, or median and interquartile range. Categorical variables are expressed as frequencies and percentages. Comparisons between groups were conducted using the independent-samples *t*-test or the Mann–Whitney U test for continuous variables, and the chi-square test for categorical variables. Univariable logistic regression analysis was initially performed to evaluate the association between each variable and the presence of ASCVD. Subsequently, multivariable logistic regression models were constructed to assess independent predictors.

A baseline model including traditional cardiometabolic risk factors (age, gender, HbA1c, LDL-C, smoking status, MetS, T2D complications) was first developed. Hepatic fibrosis scores (FIB4 and ALERT) were added to evaluate the incremental predictive value. Separately, antidiabetic therapies (iSGLT2, GLP-1 Ra, iDPP4, sulfonylurea, insulin) were included followed by a fully adjusted model incorporating both hepatic and therapeutic variables.

Regression coefficients, odds ratios, 95% confidence intervals, and *p*-values were reported. A *p*-value < 0.05 was considered statistically significant. ROC curve analyses were performed to evaluate the discriminative ability of individual predictors for the presence of ASCVD. The AUC values reported in the tables under the ROC curves therefore correspond to individual variables included in each regression model. Because of the limited sample size and the small number of patients without ASCVD, formal model-level AUC comparisons based on predicted probabilities from the full multivariable models were not performed.

## 3. Results

**Figure 1:**
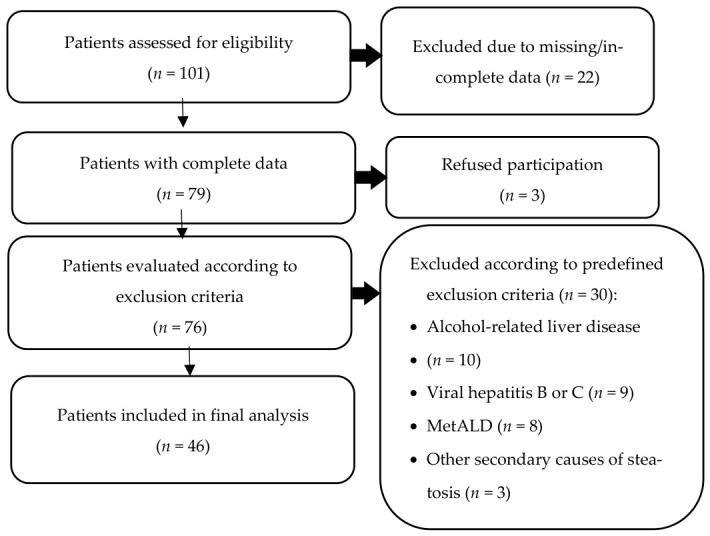
Flowchart of patient selection. Metabolic Alcohol-related Liver Disease (MetALD).

Of the 101 patients initially screened for eligibility, 22 were excluded due to incomplete data, including incomplete biological profiles, missing medication data, incomplete ASCVD assessment, or unavailable abdominal ultrasound examination. Three patients refused to participate, and 30 patients met the exclusion criteria. Therefore, 46 patients were included in the final analysis, as shown in Figure 1.

### 3.1. Cohort’s Characteristics

The patients included were 63 ± 10.36 years old, mostly females (70%), with the MetS phenotype (obesity, poor glycaemic control, HBP, abdominal circumference, and altered lipid profile).

The main characteristics of the study group include demographic, T2D complications, comorbidities, and associated medications, and are summarized in Table 1.

### 3.2. Biological Parameters

The main biological parameters of the study group include metabolic, renal, hepatic, and blood parameters, and are summarized in Table 2.

When comparing the classic and modern cardiometabolic risk factors regarding ASCVD, there was a statistically significant difference for HbA1c (8.33 ± 1.66 for ASCVD versus 7.01 ± 0.59, 95% CI 1.31 (0.11, 2.52), *p* = 0.033), BP element from MetS (*p* = 0.028), T2D complications presence (*p* = 0.028), CKD (*p* = 0.001), and for insulin treatment (*p* = 0.019). At the same time, no statistically significant differences were obtained for LDL-C, age, gender, smoking status, MetS elements, neuropathy, retinopathy, ischemic heart disease, PAD, FIB-4 score, Alert score, iSGLT2 treatment and GLP-1 Ra treatment.

**Figure 2:**
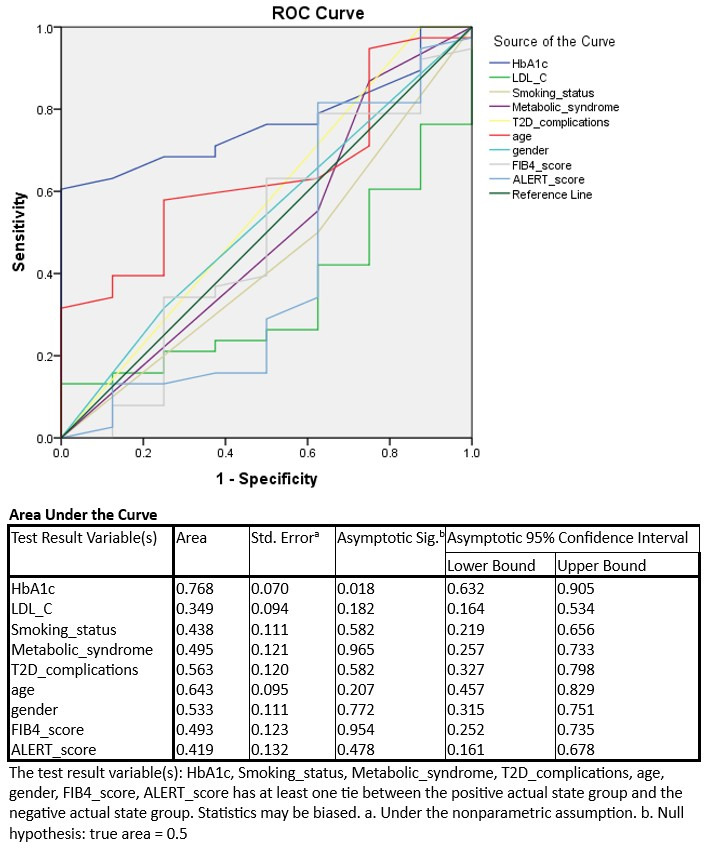
Predictor-level ROC curves for variables included in model 1 and model 2. Model 1 included HbA1c, LDL-C, smoking status, metabolic syndrome, T2D complications, age, and gender. Model 2 included the variables from model 1 plus FIB-4 score and ALERT score. The AUC values shown under the ROC curves correspond to individual predictors, not to the full multivariable models. Hemoglobin A1c (HbA1c); Low-Density Lipoprotein cholesterol (LDL-cholesterol); Metabolic Syndrome (Metabolic); Type 2 Diabetes complications (T2D_co); Fibrosis-4 Index (FIB4_s); Alert Score (ALERT-s).

Regression model 1 includes HbA1c, LDL-C, smoking status, metabolic syndrome, T2D complications, age, and gender. Regression model 2 includes the elements of model 1 plus FIB-4 score and ALERT score. For both models, age is the only significant independent risk factor, OR = 1.16, 95% CI (1.01, 1.33), *p* = 0.03, while HbA1c was borderline, OR = 7.44, 95% CI 0.92, 60.39, *p* = 0.06 for model 1 and age with OR = 1.24, 95% CI (1.00, 1.54), *p* = 0.46, while HbA1c was borderline, OR = 12.04, 95% CI 0.90, 160.80, *p* = 0.06 for model 2. Predictor-level ROC analysis showed that HbA1c had the highest discriminative ability among the variables included in models 1 and 2, with an AUC of 0.77 (95% CI 0.65–0.88, *p* = 0.018). These AUC values refer to the discriminative performance of individual predictors and should not be interpreted as AUCs of the full multivariable models. The ROC curves are shown in Figure 2.

**Figure 3:**
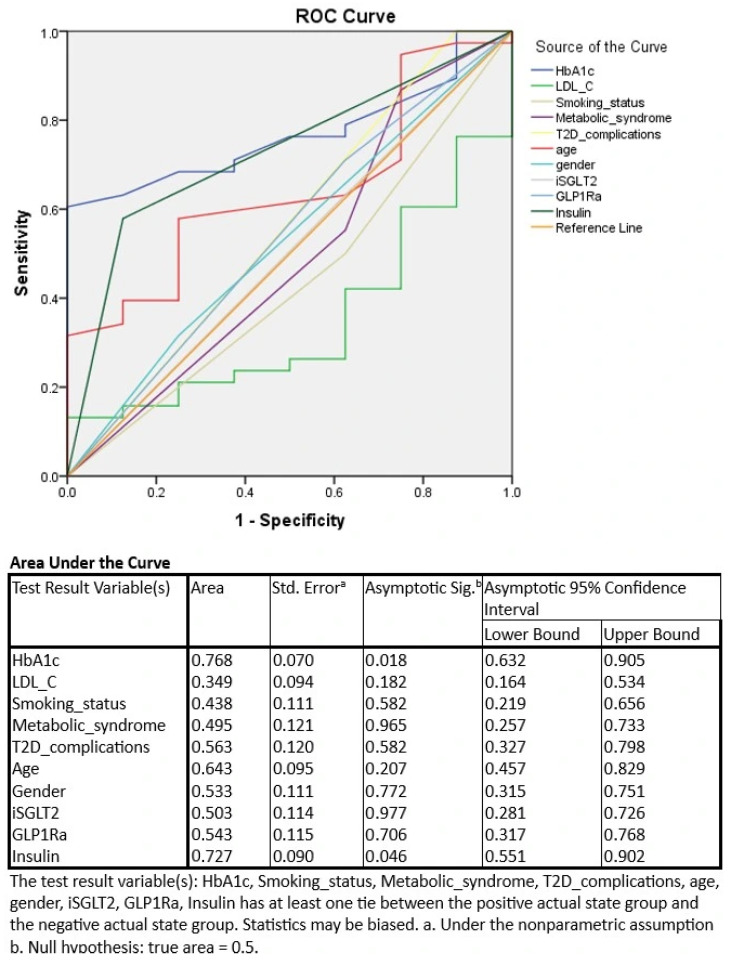
Predictor-level ROC curves for variables included in model 3. Model 3 included the variables from model 1 plus SGLT2i treatment, GLP-1 RA treatment, and insulin treatment. The AUC values shown under the ROC curves correspond to individual predictors, not to the full multivariable model. Hemoglobin A1c (HbA1c); Low-Density Lipoprotein cholesterol (LDL-cholesterol); Metabolic Syndrome (Metabolic); Type 2 Diabetes complications (T2D_co); GLP-1 receptor agonists (GLP-1 RAs); sodium–glucose cotransporter 2 inhibitors (iSGLT2).

Regression model 3 is model 1 plus SGLT2i treatment, GLP1 RA treatment and insulin treatment, age is the only significant independent risk factor, OR = 1.21, 95% CI (1.02, 1.43), *p* = 0.03. In the predictor-level ROC analysis, HbA1c showed the highest discriminative ability, with an AUC of 0.77 (95% CI 0.65–0.88, *p* = 0.018), followed by insulin treatment, with an AUC of 0.73 (95% CI 0.58–0.87, *p* = 0.046). These AUC values represent individual predictor-level discrimination and not the overall performance of model 3. The ROC curves are shown in Figure 3.

**Figure 4:**
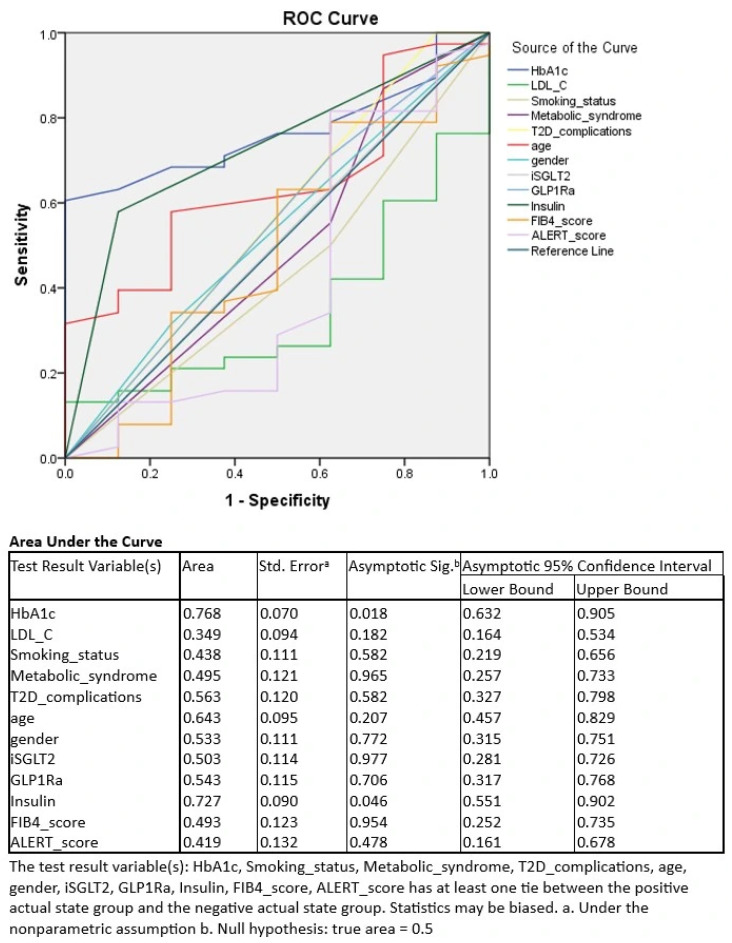
Predictor-level ROC curves for variables included in model 4. Model 4 included HbA1c, LDL-C, smoking status, metabolic syndrome, T2D complications, age, gender, FIB-4 score, ALERT score, SGLT2i treatment, GLP-1 RA treatment, and insulin treatment. The AUC values shown under the ROC curves correspond to individual predictors, not to the full multivariable model. Fibrosis-4 Index (FIB4_s); Hemoglobin A1c (HbA1c); Low-Density Lipoprotein cholesterol (LDL-cholesterol); Metabolic Syndrome (Metabolic); Type 2 Diabetes complications (T2D_co); GLP-1 receptor agonists (GLP-1 RAs); sodium–glucose cotransporter 2 inhibitors (iSGLT2).

Regression model 4, which includes model 1 elements (HbA1c, LDL-C, smoking status, metabolic syndrome, T2D complications, age, and gender) plus FIB-4 score, SGLT2i treatment, GLP1 Ra treatment, and insulin treatment. In this fully adjusted model, age was no longer independently associated with ASCVD. Predictor-level ROC analysis again identified HbA1c as the variable with the highest discriminative ability, with an AUC of 0.77 (95% CI 0.65–0.88, *p* = 0.018), followed by insulin treatment, with an AUC of 0.73 (95% CI 0.58–0.87, *p* = 0.046). These findings reflect the discriminative performance of individual predictors, not the overall discriminative performance of the full multivariable model. The ROC curves are shown in Figure 4.

## 4. Discussion

In this cross-sectional study of patients with T2D and MASLD, traditional CMRFs, particularly glycaemic control, were more strongly associated with the prevalence of ASCVD than hepatic fibrosis scores or antidiabetic therapies. Notably, hepatic markers such as FIB-4 and ALERT did not provide incremental discriminative value in this cohort.

Age showed one of the most consistent associations with ASCVD among the evaluated clinical variables, although this association was attenuated in the fully adjusted model. This finding should be interpreted cautiously, given the small sample size, the limited number of patients without ASCVD, and the risk of model overfitting. Similar findings have been reported in patients with severe obesity and MASLD, of which 21.5% of them had T2D. Age and HbA1c remained dominant predictors of vascular damage, evaluated by carotid intima-media thickness (cIMT) and pulse wave velocity (PWV), while fibrosis indices showed limited independent contribution [17]. Although age is not a modifiable risk factor, its association with ASCVD may have practical clinical relevance. In patients with T2D and MASLD, older age may reflect longer cumulative exposure to CMRFs, including hyperglycemia, hypertension, and dyslipidemia. Therefore, older patients with this phenotype may benefit from closer cardiovascular assessment and more intensive optimization of modifiable risk factors, including glycaemic control, lipid levels, blood pressure, smoking status, body weight, and kidney function. In this context, age may serve as a simple clinical marker to help identify patients who should prioritize ASCVD screening and risk-factor management.

An important clarification concerns the interpretation of the ROC analyses. The AUC values reported in the present study reflect the discriminative ability of individual predictors, such as HbA1c or insulin treatment, rather than the overall discriminative performance of the complete multivariable regression models. Therefore, these results should be interpreted as exploratory predictor-level discrimination analyses. They do not allow direct comparison of the global performance of the different multivariable models. Formal model-level ROC analyses based on predicted probabilities from each full model would be required to compare whether adding fibrosis markers or treatment variables improves overall model discrimination beyond traditional cardiometabolic risk factors. HbA1c indicated that chronic glycaemic exposure is a major determinant of vascular disease in this cohort. The mean HbA1c level of 8.1 ± 1.6% reflects suboptimal glycaemic control and a substantial metabolic burden. In addition, the long duration of T2D (mean 14 years) further supports the role of cumulative glycaemic exposure in the development of atherosclerosis. This is consistent with evidence showing that T2D and persistent hyperglycemia promote insulin resistance, systemic inflammation, and atherogenic dyslipidemia, thereby accelerating both hepatic disease progression and atherosclerosis [10,12,18].

FIB-4 was not associated with ASCVD and did not improve model performance, demonstrating limited discriminative ability in this cohort. The low variability in fibrosis risk may partly explain this, as the majority of patients were classified as low risk according to FIB-4, thereby reducing its capacity to differentiate vascular outcomes. In addition, FIB-4 is primarily designed as a screening tool for advanced liver fibrosis rather than a specific marker of vascular disease.

While several large cohort studies have demonstrated an association between elevated FIB-4 and cardiovascular outcomes, these findings were primarily observed in populations with a broader distribution of fibrosis severity and longitudinal follow-up [19,20,21,22,23]. Similarly, recent longitudinal data have independently associated higher FIB-4 values with CAC progression in individuals with MASLD. However, the reported effect sizes were modest, and FIB-4 was interpreted as a marker of underlying cardiometabolic dysfunction rather than a strong predictive tool [14]. This association was mainly observed in older individuals (>40 years old) and men, and was not significant in younger populations. In this context, FIB-4 may be more relevant for the progression of subclinical atherosclerosis rather than the presence of established disease, which may explain the lack of association observed in the present cross-sectional study of patients with advanced cardiometabolic burden and prevalent atherosclerosis.

Nevertheless, FIB-4 may still have clinical utility for identifying individuals who could benefit from more intensive cardiovascular evaluation, particularly in populations with a broader spectrum of liver disease. From a pathophysiological perspective, FIB-4 reflects not only hepatic fibrosis but also underlying systemic processes, including inflammation and metabolic dysfunction [2,15].

Antidiabetic medications, including SGLT2i and GLP-1 RA, were not independently associated with ASCVD. In contrast to our cross-sectional study, other longitudinal and interventional studies have suggested that those therapies may attenuate subclinical atherosclerosis [24]. In particular, liraglutide has shown improvement in cIMT in patients with T2D and NAFLD (MASLD) [25], potentially by reducing pro-atherogenic lipid species, such as ceramides and phospholipids, which accumulate in atherosclerotic plaques and promote lipoprotein retention and vascular inflammation, thereby supporting a potential anti-atherogenic effect [26].

Additionally, other GLP-1 RAs, such as semaglutide, have been associated with reductions in cIMT in T2D patients in real-world settings [27]. This population, like ours, was characterized by a high cardiometabolic burden, including a high prevalence of HBP, dyslipidemia, and obesity, as well as suboptimal glycaemic control and LDL-c levels above recommended targets. This longitudinal study suggests that subcutaneous semaglutide improves subclinical atherosclerosis even in high-risk populations, potentially through pleiotropic effects on atherogenic lipoproteins, inflammation, and metabolic parameters [27,28], and also showed a positive effect on hepatic steatosis. It is worth mentioning that semaglutide is recommended for MASH (F2-F3 fibrosis) without cirrhosis, based on the latest Updated Global Consensus Recommendations [13].

Dulaglutide has been shown to reduce liver fat content in patients with T2D and NAFLD (MASLD) in a randomized controlled trial, along with improvements in glycaemic control and body weight [29]. These findings further support the role of GLP-1 RAs in modulating key metabolic and hepatic pathways linked to cardiovascular risk, although their direct impact on structural atherosclerosis remains less clearly established.

In contrast, evidence regarding SGLT2i and subclinical atherosclerosis remains less consistent. While some smaller studies have suggested potential improvements in plaque volume and percent atheroma volume [30], randomized data have not consistently demonstrated a significant effect on cIMT [31]. While liraglutide was associated with a reduction in cIMT, this effect became evident only after 3 months and was less pronounced compared to empagliflozin, which induced an earlier and greater decrease in cIMT [32]. These findings suggest a potential beneficial effect of SGLT2i for enhancing endothelial function, which supports the cardioprotective properties of this class of medication even more [33].

In the present study, insulin therapy was more frequently observed in patients with ASCVD and showed moderate discriminative capacity, approximately 50% of patients were receiving insulin therapy. However, it was not independently associated with the outcome after multivariable adjustment. These findings are in the context of the high cardiometabolic burden of our cohort, characterized by long-standing T2D (14 years), suboptimal glycaemic control, and a high prevalence of complications (neuropathy 83%, mixed neuropathy 13%, while retinopathy 17%). In such settings, insulin therapy is typically introduced in patients with more advanced disease, reflecting cumulative metabolic exposure rather than exerting a direct effect on vascular pathology. Therefore, the observed association between insulin use and ASCVD is most likely explained by its role as a marker of disease severity, consistent with the broader interplay between MASLD, T2D, and cardiovascular risk.

This study has several strengths. First, it reflects a real-life clinical cohort of patients with both T2D and MASLD, a population at particularly high cardiometabolic risk. Second, the study evaluated traditional CMRFs, hepatic non-invasive fibrosis tests, and antidiabetic treatment variables within the same analytical framework. Third, the analysis focused on clinically accessible markers, such as HbA1c and FIB-4, which are widely available in routine practice. Finally, the findings highlight the potential limitations of relying on liver fibrosis scores alone for vascular risk stratification in patients with advanced cardiometabolic burden and prevalent ASCVD.

This study has several limitations. Its cross-sectional design precludes inference of causality or temporal relationships among the variables. First, the relatively small sample size (46 patients) may have limited statistical power, reducing the ability to detect weaker associations. Second, the study population was recruited from a single tertiary care center in Romania and was predominantly female, potentially introducing selection bias and limiting generalizability. The number of patients without ASCVD was also small, increasing the risk of overfitting in multivariable models with several predictors. Consequently, some estimates may be unstable, as reflected by wide confidence intervals. These findings should therefore be interpreted cautiously and require confirmation in larger prospective cohorts. In addition, the ROC analyses were performed at the predictor level. The AUC values reported for variables such as HbA1c or insulin treatment should not be interpreted as AUCs of the full multivariable models. Because of the small sample size and the limited number of patients without ASCVD, model-level AUCs derived from predicted probabilities of the complete regression models were not formally compared. Future studies with larger cohorts should evaluate full model-level discrimination and compare nested models to determine whether hepatic fibrosis markers or treatment-related variables provide incremental predictive value beyond traditional cardiometabolic risk factors. Also, the study population consisted of patients with long-standing T2D and a high cardiometabolic burden, resulting in limited variability in key parameters, particularly fibrosis scores, which likely contributed to the absence of an association with FIB-4. Finally, heart failure status and left ventricular ejection fraction were not systematically available for all participants and could not be included in the present analysis without introducing additional missing-data bias. This limited the cardiovascular characterization of the cohort and should be addressed in future prospective studies.

Hepatic fibrosis was assessed using non-invasive markers rather than imaging or histology, which may affect the precision of fibrosis stratification. ASCVD was evaluated at a single time point, preventing assessment of disease progression or treatment-related changes. The analysis of antidiabetic therapies, including insulin, SGLT2i, and GLP-1 RAs, is also subject to confounding by indication, as these treatments are more commonly used in patients with more advanced disease.

Despite these limitations, the study provides real-life data on the relative contribution of metabolic and hepatic factors in a high-risk population.

Future research should include larger prospective cohorts with longitudinal follow-up to evaluate ASCVD progression rather than focusing solely on prevalent disease. In patients with T2D and MASLD, future studies should not only assess traditional CMRFs and hepatic markers, but also evaluate how integrated treatment strategies influence cardiovascular outcomes over time. This includes the duration, timing, adherence, and intensity of pharmacological therapies, such as GLP-1 RAs, SGLT2i, lipid-lowering therapy, antihypertensive treatment, and antiplatelet therapy, as indicated. In addition, a more detailed cardiovascular characterization, including heart failure status, left ventricular ejection fraction, cardiac imaging, and functional capacity, would help identify patients who may benefit from multidisciplinary management. Cardiac rehabilitation, lifestyle intervention, structured physical activity, and optimization of pharmacological treatment may be particularly relevant in patients with overlapping MASLD, T2D, ASCVD, and heart failure. The importance of integrated pharmacological treatment and cardiac rehabilitation in patients with chronic heart failure and coronary artery disease has also been emphasized by Ciuca-Pană et al. [34].

## 5. Conclusions

In this cohort of patients with T2D and MASLD, ASCVD was associated with traditional CMRFs more than with hepatic fibrosis markers or treatment status. Among the variables studied, HbA1c showed the highest predictor-level discriminative ability, supporting the role of cumulative glycemic exposure in the development of vascular disease.

No independent relationship was observed for FIB-4. This likely reflects both the cross-sectional design and the advanced cardiometabolic profile of the population, in which atherosclerosis was already prevalent.

Overall, these results suggest that metabolic burden can be the dominant factor in atherosclerosis in this setting. At the same time, the contributions of liver-related markers and treatment effects may be more apparent in longitudinal analyses. Future prospective studies are needed to determine whether these relationships differ in earlier stages of disease or over time.

## Figures and Tables

**Table 1:** Main characteristics of the studied group.

	*n* = 46
Age, mean ± standard deviation	63 ± 10.36 years
Females, *n*, %	32, 70%
Urban settlement, *n*, %	25, 54%
T2D characteristics	
T2D duration, median, IQR	14, (8, 20) years
T2D heredity, *n*, %	28, 61%
T2D complication	
Neuropathy, *n*, %	38, 83%
Mixed neuropathy, *n*, %	6, 13%
Retinopathy, *n*, %	8, 17%
Chronic kidney disease, *n*, %	23, 50%
Ischemic heart disease, *n*, %	19, 41%
Atherosclerotic cardiovascular disease, *n*, %	38, 83%
Peripheral artery disease, *n*, %	12, 26%
Stroke, *n*, %	9, 20%
Clinical characteristics	
BMI, mean ± standard deviation	33 ± 5.53 kg/m^2^
Normoponderal, *n*, %	1, 2%
Overweight, *n*, %	10, 22%
Obesity type 1, *n*, %	19, 41%
Obesity type 2, *n*, %	10, 22%
Obesity type 3, *n*, %	6, 13%
Abdominal circumference, mean ± standard deviation	108 ± 11.46 cm
Systolic BP, mean ± standard deviation	138 ± 19.83 mmHg
Diastolic BP, mean ± standard deviation	83 ± 10.66 mmHg
Comorbidities	
HBP, *n*, %	45, 98%
Dyslipidemia, *n*, %	22, 48%
Hepatic steatosis, *n*,%	46, 100%
Metabolic syndrome, *n*, %	46, 100%
Number of MetS elements, mean ± standard deviation	4 ± 0.75
Left ventricular hypertrophy, *n*, %	23, 50%
Smoking status, *n*, %	19, 41%
T2D medication	
Metformin, *n*, %	46, 100%
GLP-1 RA, *n*, %	32, 70%
iSGLT2, *n*, %	29, 63%
DPP4i, *n*, %	3, 7%
Insulin-therapy, *n*, %	23, 50%
Sulphonylurea, *n*, %	5, 11%

T2D—type 2 diabetes; IQR—interquartile range; BMI—body mass index; BP—blood pressure; HBP—high blood pressure; GLP-1 RAs—GLP-1 receptor agonists, iSGLT2—sodium–glucose cotransporter 2 inhibitors, DPP4i—Dipeptidyl Peptidase-4 Inhibitors.

**Table 2:** Main biological parameters of the study group.

	*n* = 46
HbA1c, mean ± standard deviation	8.1 ± 1.6%
Glycemia, mean ± standard deviation	156 ± 70.61 mg/dL
Total cholesterol, mean ± standard deviation	178 ± 61.62 mg/dL
HDL cholesterol, mean ± standard deviation	43 ± 9.96 mg/dL
LDL cholesterol, median, IQR	90.37, (65.56, 122.93) mg/dL
Triglycerides, median, IQR	158.58, (117.42, 200.25) mg/dL
Creatinine, mean ± standard deviation	0.86 ± 0.25 mg/dL
eGFR, mean ± standard deviation	84 ± 21.63 mL/min/1.73 m^2^
UAC, median, IQR	42 ± 13.7 mg/dL
Urea, mean ± standard deviation	42 ± 13.70 mg/dL
FIB4, median, IQR	0.47, (0.26, 0.69)
Low risk (<1.3), *n*, %	44, 96%
Intermediate risk, *n*, %	2, 4%
High risk (>2.67), *n*, %	0
Alert, median, IQR	0.49, (0.33, 0.68)
Low risk (<0.3), *n*, %	9, 19.6%
Intermediate risk, *n*, %	23, 50%
High risk (>0.6), *n*, %	14, 30.4%

IQR—interquartile range; HbA1c—Hemoglobin A1c; LDL-cholesterol—Low-Density Lipoprotein-cholesterol; HDL-cholesterol—High Density Lipoprotein-cholesterol; FIB-4—Fibrosis-4 Index; UAC—Urinary Albumin–Creatinine ratio.

## Data Availability

The raw data supporting the conclusions of this article will be made available by the authors on request.

## Abbreviations

The following abbreviations are used in this manuscript:

ASCVD	Atherosclerotic Cardiovascular Disease
BMI	Body mass index
CAC	Coronary artery calcification
cIMT	carotid intima-media thickness
CMRFs	Cardiometabolic risk factors
CVD	cardiovascular disease
FIB-4	Fibrosis-4 index
GLP-1 RA	Glucagon-like Peptide-1 Receptor Agonists
HBP	High Blood Pressure
HDL-cholesterol	High-Density Lipoprotein cholesterol
LDL-cholesterol	Low-Density Lipoprotein cholesterol
MASH	Metabolic associated Steatohepatitis
MASLD	Metabolic dysfunction-associated steatotic liver disease
NAFLD	Non-alcoholic fatty liver disease
NIT	Non-invasive tests
PWV	pulse wave velocity
SGLT2i	Sodium–Glucose cotransporter-2 inhibitors
T2D	Type 2 Diabetes
US	Ultrasound
